# The Temptations of Physicians

**Published:** 1878-06

**Authors:** 


THE TEMPTATIONS OF PHYSICIANS.—In the admirable valedictory address of Dr. William Goodell, before the graduating class of the medical department of the University of Pennsylvania, occurs the following passage, which cannot be too deeply pondered by every one of our profession:

"Do not, oh friends. oh brothers, do not, I beseech you, be cajoled, either by the ties of friendship, by the tickle of flattery, or by the glitter of gold, into doing anything that involves the loss of self-respect, and of professional dignity; into doing anything that is not right. Have "a keen and precious hatred" of everything bad, for strong, very strong temptations will be thrown in your way, hard, very hard to resist. The honor of a family is at stake, and you will be besought to save it. A man of doubtful sanity lies a dying, and you will be asked to yield to a friendly bias in giving evidence in favor of his mental vigor and testamentary capacity. A crime has been committed, and the tears and appeals of the criminal's kin will be brought to bear on you, to give such evidence as shall tend to make him irresponsible. A patient with some organic disease, or of intemperate habits, will wish to effect an insurance on his life, and you, fearful of giving umbrage, will be sorely tempted to testify to his soundness or sobriety. You will be called upon to explain the absence of some pleasure-seeking official, by giving a certificate of illness. When summoned to courts of law as medical experts or as medical witnesses, you will find it hard to resist an unbecoming bias toward your client. Before the paint on your signs is fairly dry you will be approached by those who spurn the tender name of mother. Turn, therefore, a deaf ear to all such appeals, and swerve not a hair's breadth from strict uprightness. He who barters honor and honesty for gold gains nothing but the contempt of those who use him as their tool. Remember, besides, that you will one day answer for it, unless you keep your hands and your hearts clean, before God and man."—*Medical and Surg. Reporter.*

